# In-Hospital Mortality and Complication Rates According to Health Insurance Data in Patients Undergoing Hyperthermic Intraperitoneal Chemotherapy for Peritoneal Surface Malignancies in Germany

**DOI:** 10.1245/s10434-020-09301-z

**Published:** 2020-11-09

**Authors:** Lisa Überrück, Giorgi Nadiradze, Can Yurttas, Alfred Königsrainer, Ingmar Königsrainer, Philipp Horvath

**Affiliations:** 1 Department of General, Visceral and Transplant Surgery, Comprehensive Cancer Center, University of Tübingen, Tübingen, Germany; 2 National Center for Pleura and Peritoneum, University of Tübingen, Tübingen, Germany; 3 Department of General, Visceral and Thoracic Surgery, Feldkirch Academic Teaching Hospital, Feldkirch, Austria

## Abstract

**Background:**

Morbidity and in-hospital mortality rates of patients undergoing cytoreductive surgery and hyperthermic intraperitoneal chemotherapy in Germany are not known.

**Methods:**

From 2009 to 2018 all patients undergoing cytoreductive surgery and hyperthermic intraperitoneal chemotherapy in Germany were retrospectively analyzed regarding morbidity and in-hospital mortality rates according to nationwide hospital billing data based on diagnosis-related groups (DRG). The “failure to rescue” (FTR) index, characterizing patients who died after severe but potentially manageable complications, was calculated.

**Results:**

In total, 8463 patients were included and analyzed. Female sex predominated (1.5:1). Colonic origin of peritoneal metastasis was highest throughout all years, reaching its highest level in 2017 (55%; *n* = 563) and its lowest level in 2012 (40%; *n* = 349). Median length of hospital stay reached its maximum in 2017 at 23.9 days and its minimum in 2010 at 22.0 days. Analysis of the total FTR index showed a noticeable improvement over the years, reaching its lowest values in 2017 (9.8%) and 2018 (8.8%). The FTR index for sepsis, peritonitis, and pulmonary complications significantly improved over time. Of the 8463 included patients, 290 died during hospital stay, reflecting an in-hospital mortality rate of 3.4%.

**Conclusion:**

In-hospital mortality after cytoreductive surgery and hyperthermic intraperitoneal chemotherapy is reasonably low compared with other surgical procedures. The improvement in the FTR index reflects efforts to centralize treatment at specialized high-volume centers.

In the past, pre- or intra-operative diagnosis of peritoneal surface malignancies (PSM) prompted the interdisciplinary decision to abandon further surgical efforts, and patients underwent systemic chemotherapy with poor prognosis, mostly due to progression of peritoneal tumor implants resulting in intestinal obstruction and ultimately death. In the last couple of years, improvements in palliative chemotherapy and cytoreductive surgery (CRS) with hyperthermic intraperitoneal chemotherapy (HIPEC) were able to achieve a survival benefit for a selected subset of patients with colorectal, ovarian, appendix peritoneal metastasis, mesothelioma, and pseudomyxoma peritonei.^1^^–^17 After almost 20 years of HIPEC administration, the process of selecting appropriate patients underwent an outstanding evolution. Meanwhile, there are a variety of positive and negative prognostic factors (patient, tumor, and molecular pathological features) that influence the decision-making process to reduce postoperative morbidity and mortality rates and optimize the oncological benefit. A large number of publications have focused on the occurrence, severity, and predisposing parameters of CRS- and HIPEC-associated morbidity in recent and past literature.^18^^–^23 However, the major problem is the comparability of results, because indications, HIPEC protocols, surgical techniques, and documentation of adverse events are not uniform. A systematic review published in 2009 including 24 centers reported a major morbidity rate ranging from 12 to 52% and a mortality rate ranging from 0.9 to 5.8%.^24^ The majority of data come from countries other than Germany and often depict a selected subset of patients, thus making interpretation difficult. The true in-hospital mortality and complication rates after CRS and HIPEC in Germany are not known. Furthermore, the quality of interdisciplinary management of severe but potentially treatable complications, as assessed by the “failure to rescue” (FTR) index,^25^ is not known.

To obtain a valid insight into the quality of care of patients following CRS and HIPEC for PSM in Germany, we conducted a nationwide analysis to obtain in-hospital mortality and complication rates. Furthermore, the FTR index, characterizing those patients who died due to a severe but potentially manageable complication, was calculated.

## Patients and Methods

Data on all patients treated from January 2009 to December 2018 were obtained from the nationwide German diagnosis-related group (DRG) statistics hosted by the German Federal Statistics Office. Data management strictly followed German data protection regulations. Patients with OPS (German procedure codes) code 8-546.0 (hyperthermic intraperitoneal chemotherapy) were included, and analysis was restricted to patients with complete data records. The OPS registry is a modified version of the International Classification of Procedures in Medicine (ICPM). The procedure had to be performed in a German hospital.

Data included primary diagnoses according to ICD-10 (ICD-10-GM) classification, sex, gender distribution, length of hospital stay, performed procedures according to the respective OPS codes, in-hospital mortality, and morbidity rates. The calculated FTR index describes the proportion of patients who died during hospital stay due to a severe but potentially treatable complication. The following severe complications were included according to their respective ICD-10-GM codes and analyzed: sepsis, myocardial infarction, acute pulmonary embolism, pneumonia, peritonitis, anastomotic insufficiency, and acute gastrointestinal bleeding.

Separate calculations of the FTR index were performed for pulmonary and bleeding complications. The FTR index (pulmonary) included the following diagnoses according to their respective ICD-10-GM codes: respiratory insufficiency, acute pulmonary embolism, pneumonia, pleural effusion, and ventilation > 24 h. The FTR index (bleeding) included the following diagnoses according to their respective ICD-10-GM codes: acute bleeding anemia, disseminated intravascular coagulation, and transfusion of more than six packed red blood cells.

### Statistical Analysis

Statistical analysis was performed using IBM SPSS software (version 25.0, IBM SPSS Inc., Chicago, IL, USA). All *p*-values were two-tailed, and a probability value of *p* < 0.05 was considered statistically significant.

## Results

From January 2009 to December 2018, a total of 8463 patients underwent CRS and HIPEC in German hospitals. In 2017, the largest number of patients was treated (*n* = 1159). There was a predominance of female sex (1.5:1 or 5132:3331 patients).

Colonic origin (C18 in ICD-10-GM) of PSM was highest throughout all the years, reaching its highest level in 2017 (55%; *n* = 563) and its lowest level in 2012 (40%; *n* = 349). The proportion of gastric origin (C16 ICD-10-GM) remained stable over the years (12–18%). The proportion of females with ovarian origin (C56 ICD-10-GM) significantly declined over the years from 25% (*n* = 98) in 2009–9% (*n* = 89) in 2018. The fourth greatest cause of PSM was peritoneal mesothelioma (C45 ICD-10-GM) with a stable proportion of 5–7% of patients per year.

The surgical procedures most frequently performed were omentectomy (47%; *n* = 3979), cholecystectomy (34%; *n* = 2892), and colonic and rectal resections (43%; *n* = 3669) (Table 1). Minor hepatic resections (8–13%) and removal of the pancreatic tail (2–4%) were performed more seldomly. In total, 24% (*n* = 2050) of the 8463 patients returned to the operating room (OR) with the highest rate in 2015 (27%) and with the lowest rate in 2010 (20%), without reaching statistical significance when comparing 2018 and the previous years (Table 1).

**Table 1:** Patient-and treatment characteristics

Parameter/year	2018	2017	2016	2015	2014	2013	2012	2011	2010	2009
Number of patients (*n*)	1084	1159	1015	1018	885	822	861	699	515	405
Sex (female) *n* (%)	630 (58)	656 (57)	596 (59)	600 (59)	534 (60)	511 (62)	581 (67)	437 (63)	335 (65)	252 (62)
Tumor etiology, *n* (%)										
Colonic	563 (54)	665 (55)	508 (49)	500 (49)	429 (48)	373 (45)	349 (40)	264 (38)	208 (41)	165 (42)
Rectal	30 (3)	60 (5)	52 (5)	55 (5)	45 (5)	36 (4)	33 (4)	27 (4)	15 (3)	14 (3)
Small bowel	26 (2)	29 (3)	21 (2)	26 (3)	25 (3)	16 (2)	15 (2)	11 (2)	10 (2)	3 (1)
Gastric	185 (18)	185 (18)	169 (16)	145 (14)	141 (16)	126 (15)	145 (17)	125 (18)	86 (17)	46 (12)
Ovarian	89 (9)	98 (8)	132 (13)	140 (14)	119 (13)	140 (17)	183 (21)	148 (22)	109 (22)	98 (25)
Mesothelioma	55 (5)	62 (5)	65 (6)	73 (7)	49 (6)	52 (6)	60 (7)	51 (7)	36 (7)	25 (6)
CUP	27 (2)	38 (3)	35 (3)	28 (3)	26 (3)	24 (3)	23 (3)	24 (3)	18 (3)	11 (3)
Pancreatic	11 (1)	23 (2)	16 (2)	13 (1)	6 (1)	9 (1)	15 (2)	4 (1)	0 (0)	9 (2)
Others	58 (5)	50 (4)	46 (5)	49 (5)	45 (5)	47 (6)	53 (6)	33 (5)	21 (4)	18 (4)
Surgical procedures, *n* (%)										
Colon/rectal resection	508 (47)	570 (49)	468 (46)	494 (49)	416 (47)	394 (48)	298 (34)	251 (36)	159 (31)	111 (27)
Omentectomy	503 (46)	508 (44)	485 (48)	525 (52)	391 (44)	323 (39)	440 (51)	357 (51)	266 (52)	181 (45)
Cholecystectomy	376 (35)	425 (37)	379 (37)	376 (37)	303 (34)	297 (36)	252 (29)	201 (29)	170 (33)	113 (28)
Minor LR	119 (11)	140 (12)	118 (12)	107 (11)	89 (10)	102 (12)	102 (12)	89 (13)	58 (11)	33 (8)
PT resection	22 (2)	46 (4)	29 (3)	24 (2)	33 (4)	36 (4)	30 (3)	24 (3)	15 (3)	9 (2)
Splenectomy	165 (15)	209 (18)	173 (17)	186 (18)	134 (15)	155 (19)	158 (18)	138 (20)	110 (21)	70 (17)
Stoma	189 (17)	218 (19)	172 (17)	211 (21)	134 (15)	147 (18)	148 (17)	103 (15)	58 (11)	58 (14)
Tracheostoma	15 (1)	21 (2)	21 (2)	17 (2)	16 (2)	19 (2)	15 (2)	6 (1)	5 (1)	8 (2)
2nd look	36 (3)	26 (2)	25 (2)	32 (3)	30 (3)	28 (3)	22 (3)	26 (4)	22(4)	15 (4)
Back to OR	249 (23)	288 (25)	263 (26)	270 (27)	213 (24)	189 (23)	193 (22)	175 (25)	105 (20)	105 (26)
Median LOS (days)	22.2	23.7	22.8	23.3	22.9	23.5	23	22.4	22	22.1
In-hospital mortality, *n* (%) (*p* value*)	30 (2.7)	28 (2.4) **(0.6)**	35 (3.5) **(0.36)**	37 (3.7) **(0.25)**	31 (3.5) **(0.34)**	35 (4.3) **(0.07)**	36 (4.2) **(0.08)**	30 (4.3) **(0.08)**	14 (2.7) **(0.95)**	14 (3.5) **(0.48)**

Bold values indicate significant at *p* < 0.05

*CUP* cancer of unknown primary, *LR* liver resection, *LOS* length of hospital stay, *OR* operating room, *PT* pancreatic tail

In 17% (*n* = 1433) of the patients, a protective or a permanent stoma was created with the highest rate in 2015 (20%) and the lowest rate in 2010 (11%).

Median length of hospital stay did not differ substantially throughout the years and at 23.9 days it reached its maximum in 2017, and its minimum in 2010 at 22.0 days.

Analysis of the total FTR index showed a noticeable improvement over the years, reaching the lowest values in 2017 (9.8%) and 2018 (8.8%) without reaching statistical significance between 2018 and the previous years (Table 2).

**Table 2:** Cumulative FTR index from 2009 to 2018 for severe but potentially treatable complications

FTR-index in % (*n*) (*p*-value)	2018	2017	2016	2015	2014	2013	2012	2011	2010	2009
Sepsis	14.7 (11)	21.4 (19) (0.27)	24.7 (19) (0.12)	29.3 (22) (0.03*)	20.3 (13) (0.38)	32.0 (15) (0.02*)	18.2 (8) (0.61)	35.5 (11) (0.01*)	18.8 (3) (0.68)	21.7 (5) (0.42)
MI	0 (0)	0 (0)	0 (0)	0 (0)	–	0 (0)	–	0 (0)	–	–
APE	10.8 (4)	10.5 (4) (0.96)	20.1 (5) (0.28)	14.0 (6) (0.67)	20.8 (5) (0.28)	15.0 (3) (0.64)	17.7 (3) (0.48)	0 (0)	23.1 (3) (0.27)	0 (0)
Pneumonia	21.6 (8)	17.1 (7) (0.61)	30.1 (8) (0.41)	30.4 (7) (0.44)	15.0 (3) (0.54)	36.8 (7) (0.22)	15.8 (6) (0.51)	0 (0)	0 (0)	0 (0)
Peritonitis	6.6 (11)	7.5 (14) (0.73)	11.0 (15) (0.17)	11.6 (18) (0.11)	15.3 (13) (0.03*)	21.8 (22) (0.0002*)	12.6 (12) (0.09)	21.1 (16) (0.0008*)	21.2 (7) (0.0007*)	28.1 (9) (0.0002*)
AI	6.1 (13)	5.2 (13) (0.70)	11.7 (24) (0.04*)	6.1 (13) (0.97)	4.8 (8) (0.59)	12.8 (16) (0.03*)	8.6 (10) (0.37)	13.4 (11) (0.03*)	3.4 (3) (0.35)	10.7 (6) (0.22)
AGB	11.5 (3)	28.6 (4) (0.17)	0 (0)	16.7 (3) (0.62)	40.0 (4) (0.053)	0 (0)	0 (0)	0 (0)	–	–
FTR % (*n*) (total)	8.8 (50)	9.8 (61)	14.5 (71)	13.0 (69)	12.4 (46)	19.5 (63)	12.3 (39)	15.8 (38)	9.9 (16)	16.0 (20)
*p*-Value (total)		0.27	0.84	0.59	0.46	0.34	0.44	0.52	0.16	0.58

Bold values indicate significant at *p* < 0.05

*AGB* acute gastrointestinal bleeding, *AI* anastomotic insufficiency, *APE* acute pulmonary embolism, *FTR* failure to rescue, *MI* myocardial infarction

— no reports

Especially the management of patients suffering from sepsis and peritonitis improved over the study period. In the year 2018, the FTR index for sepsis was 14.7% and statistically significantly improved as compared with previous years [2011: 35.5% (*p* = 0.01); 2013: 32.0% (*p* = 0.02) and 2015: 29.3% (*p* = 0.03)]. In the year 2018, the FTR index for peritonitis was 11% and also statistically significantly improved when compared with previous years [2009: 28.1% (*p* = 0.0002); 2010: 21.2% (*p* = 0.0007); 2011: 21.1% (*p* = 0.0008); 2013: 21.8% (*p* = 0.0002) and 2014: 15.3% (*p* = 0.03)] (Table 2).

Total rate of anastomotic insufficiency (AI) was 17.9% (*n* = 1515) with a statistically significant upward trend from 2009 (*p* = 0.0009), 2010 (*p* = 0.042), 2011 (*p* < 0.00001), 2012 (*p* < 0.00001), and 2013 (*p* = 0.0005) to 2017 with the highest AI rate (21.4%; *n* = 248). Nevertheless, the management and consecutive FTR index for patients with AI improved and were seen to be statistically significant when comparing the year 2018 (FTR index 6.1%) with the years 2016 (FTR index 11.7%; *p* = 0.04), 2013 (FTR index 12.8%; *p* = 0.03), and 2011 (FTR index 13.4%; *p* = 0.03) (Table 2).

The FTR index depicting pulmonary complications showed the same trend with the lowest values in 2017 (7.5%) and in 2018 (7.3%). In particular, intensive care unit (ICU) management of patients suffering from respiratory insufficiency showed the most statistically significant improvement when comparing 2018 with the previous years (Table 3).

**Table 3:** FTR-index for pulmonary complications

FTR-index in % () (*p*-value)	2018	2017	2016	2015	2014	2013	2012	2011	2010	2009
RI	15.4 (15)	15.5 (13) (0.98)	11.4 (23) (0.016*)	12.5 (24) (0.006*)	12.8 (20) (0.006*)	12.4 (17) (0.01*)	14.0 (14) (0.006*)	16.1 (10) (0.003*)	12.5 (6) (0.06)	18.5 (5) (0.008*)
APE	10.8 (4)	10.5 (4) (0.96)	20.1 (5) (0.28)	14.0 (6) (0.67)	20.8 (5) (0.28)	15.0 (3) (0.64)	17.7 (3) (0.48)	0 (0)	23.1 (3) (0.27)	0 (0)
Pneumonie	21.6 (8)	17.1 (7) (0.61)	30.1 (8) (0.41)	30.4 (7) (0.44)	15.0 (3) (0.54)	36.8 (7) (0.22)	15.8 (6) (0.51)	0 (0)	0 (0)	0 (0)
PE	5.2 (15)	6.3 (20) (0.54)	9.0 (23) (0.07)	10.4 (29) (0.018*)	8.5 (17) (0.14)	36.8 (17) (0.19)	8.9 (18) (0.10)	10.8 (17) (0.02*)	4.6 (4) (0.83)	12.6 (10) (0.018*)
Ventilation > 24 h	9.8 (20)	9.0 (21) (0.80)	11.1 (24) (0.64)	10.1 (23) (0.90)	10.2 (19) (0.89)	14.3 (25) (0.17)	11.7 (17) (0.55)	18.8 (22) (0.02*)	10.5 (9) (0.85)	7.1 (5) (0.01*)
FTR % (*n*) (total)	7.3 (62)	7.5 (65)	11.5 (83)	11.7 (89)	10.9 (64)	12.4 (68)	10.6 (58)	13.1 (49)	8.9 (22)	10.5 (20)
*p*-Value (total)		0.88	0.004*	0.002*	0.018*	0.001*	0.44	0.13	0.34	0.13

Bold values indicate significant at *p* < 0.05

*APE* acute pulmonary embolism, *PE* pleural effusion, *RI* respiratory insufficiency

Total FTR index for bleeding complications did not show significant differences between years, but the amount of transfused packed red blood cells impacted the FTR index with the cut-off of 6 (FTR index: ≤ 6: 8% vs. ≥ 6: 15%) and was statistically significant only in 2013 (*p* = 0.03) as compared with 2018 (Table 4).

**Table 4:** FTR-index for bleeding complications

FTR-rate in % (*n*) (*p*-value)	2018	2017	2016	2015	2014	2013	2012	2011	2010
ABA	4.8 (21)	4.3 (21) (0.71)	5.8 (26) (0.50)	6.3 (28) (0.34)	5.9 (23) (0.49)	7.3 (30) (0.12)	6.2 (25) (0.37)	6.5 (24) (0.29)	4.4 (10) (0.80)
DIC	0 (0)	16.7 (5)	0 (0)	16.7 (4)	16.7 (4)	0 (0)	0 (0)	31.3 (5)	0 (0)
≥ 6 PRBC	20.0 (4)	16.7 (4) (0.77)	23.1 (6) (0.80)	18.5 (5) (0.89)	16.7 (3) (0.79)	24.0 (6) (0.74)	8.3 (3) (0.20)	0 (0)	0 (0)
FTR % (n) (total)	**5.3 (25)**	**5.5 (30)**	**6.4 (32)**	**7.4 (37)**	**6.9 (30)**	**8.0 (36)**	**6.1 (28)**	**7.1 (29)**	**4.4 (10)**
*p*-Value (total)		**0.85**	**0.44**	**0.17**	**0.30**	**0.09**	**0.58**	**0.25**	**0.60**

Bold values indicate significant at *p* < 0.05

*ABA* acute bleeding anemia, *DIC* disseminated intravascular coagulation, *PRBC* packed red blood cell

Acute postoperative kidney failure (N17 ICD10-GM) occurred in 7% (*n* = 628) of all patients with a cumulative FTR index of 20% (*n* = 127). The highest failure rate was noted in 2012 (33%; *n* = 10) and the lowest in 2018 (8%; *n* = 10). Therapeutic management statistically significantly improved as seen from a comparison of 2018 with the previous years (for example, 2012 and 2013; *p* < 0.0001).

Of the 8463 included patients, 290 died during hospital stay, reflecting an in-hospital mortality rate of 3.4% with the highest rate in 2011 (4.3%; *n* = 30) and the lowest in 2017 (2.4%; *n* = 28) (Table 1). There was a trend towards reduced in-hospital mortality when comparing 2018 with 2013 (*p* = 0.07), 2012 (*p* = 0.08), and 2011 (*p* = 0.08), but without reaching statistical significance.

## Discussion

Since 2009, a steadily rising number of patients have undergone CRS and HIPEC for PSM of gastrointestinal and gynecological primary tumors in Germany. The most frequently applied criticism is that it is an aggressive surgical procedure associated with high morbidity and mortality rates and can therefore delay start of adjuvant systemic chemotherapy. Apart from this, the performance of CRS and HIPEC was, especially for colorectal cancer, critically scrutinized following the negative PRODIGE-7, PROPHYLOCHIP, and COLOPEC trials. On the other hand, Van Driel and coworkers demonstrated for ovarian cancer and cisplatin-based HIPEC a longer recurrence-free survival and overall survival than for surgery alone, without leading to a higher rate of side-effects.^9^ Despite the strong rationale and evidence for CRS and HIPEC in ovarian cancer, the numbers of patients are steadily decreasing in Germany, mainly due to the negative German guideline recommendation for administration of HIPEC in 2013.^26^

Apart from the oncological benefit, the true impact of the surgical and chemotherapeutic procedures on nationwide in-hospital morbidity and mortality rates is not known, even though in the past a large number of publications have focused on the occurrence, severity, and predisposing parameters of CRS- and HIPEC-associated morbidity.^18^^–^23 However, the major problem is comparability of results, because indications, HIPEC protocols, surgical technique, and documentation of adverse events are not uniform in Europe and the USA. To gain an insight into the current medical care situation of PSM patients in Germany, we performed a nationwide analysis of in-hospital mortality and morbidity rates. As a surrogate parameter for perioperative management quality, the FTR index, as described earlier,^27^ was calculated and analyzed.

Complete datasets of 8463 patients were analyzed, and we observed an improvement in the FTR index from 2009 to 2018 with the lowest index (8.8%) in 2018, but without reaching statistical significance when comparing it with the previous years. In particular, FTR indices for sepsis, peritonitis, pulmonary complications, and acute postoperative kidney failure significantly improved over time. These results suggest that the management of severe but potentially treatable complications has been optimized throughout the years. This phenomenon may be attributed to a stricter patient selection process than in earlier years and a steady improvement in postoperative ICU management with evolving options, especially antibiotic treatment of peritonitis-related septic conditions. A retrospective, observational study over 12 years including 101,064 patients with severe sepsis in Australia and New Zealand showed a reduction in mortality in the “surgical admission” subgroup from 25.2% in 2000 to 12.7% in 2012.^28^ The same trend was observed in nonseptic ICU patients, so the authors hypothesized that overall changes in ICU practice rather than the management of sepsis explain most of their findings.

With regard to the centralization of PSM management, the results of a French retrospective cohort, multicentric study show that the PCI and the performing center were statistically significantly linked to increased postoperative morbidity. In that study, centers were classified as experienced (> 7 years of practice) or inexperienced (< 7 years of practice).^29^ The data strongly suggest that administration of CRS and HIPEC should be centralized in high-volume centers to guarantee a good patient selection process, surgical technique, and complication management. Unfortunately, the analyzed dataset did not indicate the years of experience the respective center has with PSM treatment, and thus no further conclusions can be drawn.

The quality of the patient selection algorithm, preoperative patient conditioning, and the surgical technique itself are mainly reflected in the rate of reoperation during hospital stay. Data show that, in total, 24% of all patients returned to the OR with the highest rate seen in 2015 (27%) and the lowest rate in 2010 (20%). These rates are generally in line with those of other authors.^30^,^31^ On the other hand, in a recently published retrospective cohort study from the USA comparing high-risk surgical oncology procedures with CRS and HIPEC, the authors showed a reoperation rate after CRS and HIPEC of only 6.8%. Patients after esophagectomy had the highest reoperation rate (14.4%).^32^ Another retrospective analysis from the Netherlands showed a total reoperation rate of 16%.^33^ Patients who had all three preoperatively identified risk factors [PSS (prior surgical score) > 1, positive smoking history, and Eastern Cooperative Oncology Group (ECOG) score > 1] had a reoperation rate of 45.5%. Our dataset did not specify the reason for reoperation, so that it is possible that minor surgical procedures contributed to the reoperation rate. In another German retrospective analysis, the main reasons for reoperation were AI, fascial rupture, and pancreatitis.^34^ In our presented data, the total AI rate was 17.9%, namely higher than in recent literature, which suggests AI rates between 8% and 12%.^35^^–^39 These elevated AI rates may also explain the slightly increased reoperation rate. Nevertheless, this is the only study presenting nationwide data without any study-related selection bias. Furthermore, the rate of iterative CRS and HIPEC procedures within the study population is not known. It is presumed that repeated procedures are associated with a major late complication rate that is twice as high.^40^ The AI rate in the study by Bekhor and coworkers was 8% for primary CRS and HIPEC and 14% for repeated procedures.^40^

The stoma creation rate was 17%, which is in line with other large retrospective analyses from the USA and Israel.^41^,^42^ Doud and colleagues showed that, in their cohort, only 26% of potentially reversible stomata were indeed reversed.^41^ The main reasons were tumor progression or death. Furthermore, stoma reversal was associated with 27.9% of Clavien–Dindo III/IV morbidity. In another US database analysis, the presence of a stoma was associated with a higher 30-day readmission rate.^43^ These data show that, during CRS and HIPEC, stoma creation should be limited to ultralow rectal resections because it has been shown that addition of HIPEC does not impact AI rates and morbidity is elevated during stoma reversal.

These nationwide data showed a total in-hospital mortality rate of 3.4%, which is in line with the international literature^44^^–^46 and substantially lower than for surgical procedures for pancreatic (10.1%),^47^ esophageal cancer (9.5%),^47^ and major liver resection (16.2%)^48^ in Germany. An NSQIP Database analysis of 1822 patients after CRS and HIPEC showed an overall 30-day mortality of 1.1%, but since crucial treatment- and patient-specific parameters were not mentioned, data must be interpreted with care. In that study, 30-day mortality rates for Whipple’s procedure, right lobe hepatectomy, esophagectomy, and trisegmental hepatectomy were 2.5%, 2.9%, 3.0%, and 3.9%, respectively. On the other hand, 30-day mortality rates following oncologic colorectal surgery were 5%^49^ and 5.9%^50^ in studies from France and the USA, respectively. German data indicated an elevated in-hospital mortality rate of 7.5% following colorectal cancer surgery.^47^ These national and international data should overturn the presumption that CRS and HIPEC is associated with higher morbidity and mortality rates as compared with other high-risk and even low-risk surgical oncology procedures. This misperception becomes even more evident when it is known that approximately 60,000 patients in the USA are diagnosed with PSM every year, but in 2015 only 1000 CRS and HIPEC procedures were performed.^32^

Limitations of this study are surely its retrospective character and the fact that the occurrence of a single severe complication cannot be inevitably linked to the patient’s death, even though it is common practice in literature.^27^ Furthermore, important patient- and treatment-specific parameters, such as the HIPEC regime and compound, the peritoneal cancer index (PCI), and the completeness of the cytoreduction (CC) score, are missing. Moreover, the level of experience of all contributing centers is not known. Nevertheless, these nationwide data show that CRS and HIPEC is not associated with an elevated mortality rate by comparison with other high-risk surgical oncology procedures, and improvement of FTR indices (especially for sepsis and peritonitis) was linked to decreased in-hospital mortality. Centralization of PSM treatment in high-volume centers is highly recommended to further improve short- and long-term outcomes. Last but not least, these data may help physicians overcome their reluctance to refer patients, especially those with peritoneal metastases of ovarian cancer, to centers of excellence for evaluation for CRS and HIPEC.
